# Orthogonal NGS for High Throughput Clinical Diagnostics

**DOI:** 10.1038/srep24650

**Published:** 2016-04-19

**Authors:** Niru Chennagiri, Eric J. White, Alexander Frieden, Edgardo Lopez, Daniel S. Lieber, Anastasia Nikiforov, Tristen Ross, Rebecca Batorsky, Sherry Hansen, Va Lip, Lovelace J. Luquette, Evan Mauceli, David Margulies, Patrice M. Milos, Nichole Napolitano, Marcia M. Nizzari, Timothy Yu, John F. Thompson

**Affiliations:** 1 Claritas Genomics, Cambridge MA, USA; 2 Harvard Medical School, Boston MA, USA; 3 Boston Children’s Hospital, Boston MA, USA

## Abstract

Next generation sequencing is a transformative technology for discovering and diagnosing genetic disorders. However, high-throughput sequencing remains error-prone, necessitating variant confirmation in order to meet the exacting demands of clinical diagnostic sequencing. To address this, we devised an orthogonal, dual platform approach employing complementary target capture and sequencing chemistries to improve speed and accuracy of variant calls at a genomic scale. We combined DNA selection by bait-based hybridization followed by Illumina NextSeq reversible terminator sequencing with DNA selection by amplification followed by Ion Proton semiconductor sequencing. This approach yields genomic scale orthogonal confirmation of ~95% of exome variants. Overall variant sensitivity improves as each method covers thousands of coding exons missed by the other. We conclude that orthogonal NGS offers improvements in variant calling sensitivity when two platforms are used, better specificity for variants identified on both platforms, and greatly reduces the time and expense of Sanger follow-up, thus enabling physicians to act on genomic results more quickly.

High throughput sequencing has transformed the landscape of clinical genetics, enhancing our ability to decipher and treat rare human diseases with underlying genetic causes. There are many examples of patients ending diagnostic odysseys and benefitting from a broad, unbiased examination of their genome^1^,^2^,^3^,^4^,^5^,^6^,^7^. While some advocate whole genome sequencing, the costs of generating, analyzing, interpreting, and confirming the accuracy of such data makes it currently impractical for routine use. The great bulk of clinical sequencing is currently performed as whole exome sequencing (WES), representing the best compromise among cost, completeness, accuracy, and timeliness. WES focuses efforts on the most clinically-relevant and interpretable regions of the genome, providing excellent diagnostic value at a reasonable cost to the patient and health care system.

Each next generation sequencing (NGS) platform has its own strengths and weaknesses. Raw base-calling error rates are typically 0.5–1% but can range much higher^8^,^9^. Because of these high error rates, the American College of Medical Genetics (ACMG) practice guidelines^10^ recommend that orthogonal or companion technologies should be used to ensure that variant calls are independently confirmed and thus accurate. This has generally been carried out using Sanger sequencing but this is a relatively manual process that does not scale well for genome-wide studies.

In addition to the base-calling errors, WES performance is also impacted by the effectiveness of the method used to target DNA. Many comparisons of exome offerings have been carried out^11^,^12^,^13^,^14^,^15^,^16^,^17^,^18^,^19^ but the rapid turnover of the underlying methods makes such studies outdated very quickly. Furthermore, published studies have tended to focus on comparisons between platforms rather than how multiple methods might be used productively in tandem.

In this report, we describe a strategy for rapidly generating high quality exome variant calls by leveraging orthogonal and independent NGS technologies for both selection and sequencing of DNA. Many thousands of variants can be simultaneously called and confirmed at a genomic scale. We show that these methods provide high quality and complete exome data compatible with the needs of clinical diagnostics, enhancing the ability of patients to get answers in a timely manner.

## Methods

Purified NA12878 DNA for sequencing was obtained from both the National Institute of Standards and Technology (NIST, Gaithersburg, MD) and Coriell Institute for Medical Research (Camden, NJ). Clinical sequencing was performed with DNA isolated using an Autogen FlexStar for blood volumes greater than 2 ml and a QiaCube for lower blood volumes and for saliva. For Illumina-based sequencing, DNA was targeted using the Agilent Clinical Research Exome kit for hybridization capture and then made into libraries using the QXT library preparation kit based on manufacturer’s recommendations. These were then sequenced on either a MiSeq or NextSeq (with version 2 reagents) as recommended by Illumina. Both MiSeq and NextSeq data underwent alignment, cleaning, and variant calling according to GATK best practices^20^. Tools and versions used for analysis include BWA-mem (0.7.10-r789), sambamba (v0.4.7), CalculateHSMetrics.jar (1.84(1332)), picard.jar (Version: 1.124(69ecf101f612fdc0f3d555aa2d3cc0b1ea193c68_1415030499)), IGVTools_2.3.36, bcl2fastq (2.16), bedtools2-2.19.1, and samtools-1.2. For many analyses, final Illumina variant calls were also subjected to minimum depth and quality thresholds of DP > 8 and GQ > 20 chosen to minimize loss of true variants while filtering out as many false positives as possible^21^. Variants filtered out by DP or GQ are retained but classified as NoPass calls for further evaluation.

DNA sequenced on the Ion Torrent Proton was targeted using the Life Technologies AmpliSeq Exome kit as directed by the manufacturer with libraries prepared on the OneTouch system. Libraries were then sequenced on the Ion Proton^TM^ system with HiQ polymerase. Read alignment, cleaning, and variant calling was performed using Torrent Suite v4.4 followed by application of additional custom filters to remove strand-specific errors and recurrent false positives generated from over 6000 Proton exomes sequenced by Claritas Genomics (filters to be made available through Life Technologies in future software updates).

Variant calls from Illumina and Ion Torrent were combined using a custom algorithm (Combinator) developed by Claritas for integrating multi-platform VCF files. Briefly, variants are compared across platforms and grouped into classes based on a set of attributes including whether the variant is a SNP or indel, whether the variant call and zygosity match between both platforms, and whether the variant site is well-covered in each platform. To assess the accuracy of each variant class, the algorithm was applied to NA12878 orthogonal sequencing data and compared to the NIST Genome In A Bottle NA12878 truth set (v2.17). Some analyses were also carried out using truth set v2.19. A positive predictive value (PPV) was calculated for each variant class and used for subsequent applications of orthogonal sequencing to guide variant interpretation.

## Results

To assess the performance of different exome sequencing strategies, we used the reference sample NA12878 from HapMap in conjunction with the gold standard reference call set maintained by NIST^22^. Sequencing libraries were generated in parallel using both the oligo-based Agilent SureSelect Clinical Research Exome (CRE) and the amplification-based AmpliSeq Exome Kit. Three independent libraries were made using each method. These libraries were sequenced on the NextSeq (CRE), MiSeq (CRE), and Proton (AmpliSeq) platforms to an average coverage of 125×, 46× and 133×, respectively. Variants were called and then compared to the NIST reference for completeness and accuracy.

We chose as our analytic region all RefSeq coding exons (CDS) as well as 10 bp on both sides of each exon in order to capture all clinically-relevant regions including splicing mutations (~37.6 Mb, Table 1). After intersection with the 2.2 Gb NA12878 NIST v2.17 reference truth set, this yielded a final analytic target of 28.2 Mb. Both capture methods include nearly all the RefSeq sequences. The Agilent CRE design specifically targets 99.7% of this analytic region (and 97.6% of RefSeq overall) while the AmpliSeq Exome design targets 97.9% of this analytic region (and 95.6% of RefSeq overall). The overall intersection of RefSeq, NIST, CRE and AmpliSeq yields 27.6 Mb in common.

**Figure 1:**
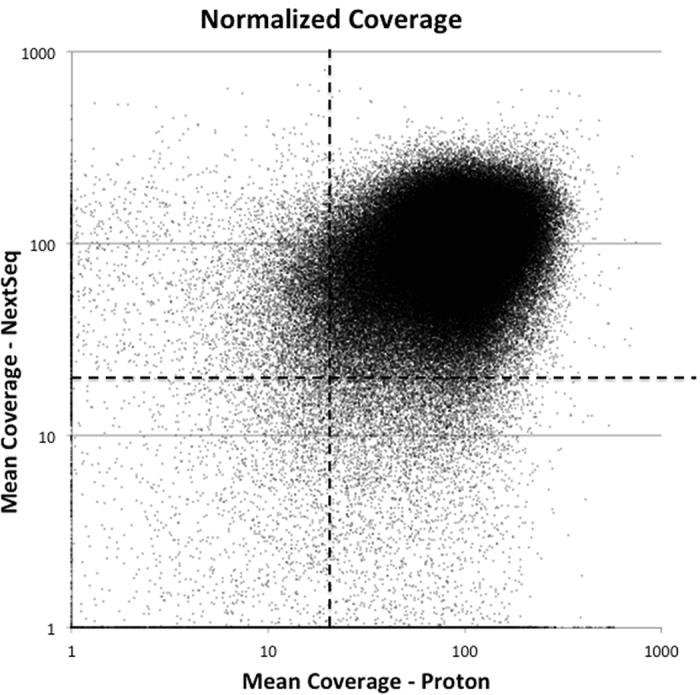
Comparison of per-exon coverage achieved on NextSeq and Proton platforms. Mean coverage for each exome was normalized to 100×. Coverage for each exon was plotted on a log scale with exons having no reads changed to 1× for plotting. Dashed lines show 20× coverage for each platform.

First, we analyzed how well each sequencing approach covered our analytic target. For comparison purposes, NextSeq and Proton data was numerically normalized to a mean depth of 100×. In Fig. 1, mean coverage on both platforms for each exon is plotted for all 187,475 exons in our analytic region as a function of sequencing method. Exons with no coverage were adjusted up to 1× in order to allow log-log plotting. The graph was split into four quadrants based on mean 20× coverage. The great majority of exons (>90%) were covered by at least 20 reads by both platforms. 4327 (2.3%) exons failed to achieve at least 20× coverage on both platforms with 2253 (1.2%) of these having less than 10× coverage on both platforms. More than 8% of exons were well covered (>20× mean coverage) by one platform but not the other (4.7% or 8892 with >20× coverage on NextSeq only and 3.7% or 6973 with >20× coverage on Proton only). Many of the exons found on only the NextSeq or only the Proton are difficult to sequence and thus NIST has not included them in their reference. Because they are not in NIST, they do not impact the apparent sensitivity listed in Table 2. Thus, use of two orthogonal platforms improves the orthogonal sensitivity ~3–4% relative to the use of one platform alone. This estimate is based on the number of exons where variants can be detected on only one platform.

To better understand which exons are poorly covered, the impact of GC-content on coverage was examined (Supplementary Figure 1). After normalization of coverage on both platforms to 100×, the number of exons that did not achieve 20× coverage in each platform is shown as a function of GC content. Neither platform performs as well at the extremes of GC-content though the Proton tends to be better with AT-rich exons and the NextSeq with GC-rich exons. Both platforms have better coverage with 40–70% GC-content.

Next, we analyzed how each individual platform performed with respect to calling accuracy. Within the complete analytic region as well as some representative smaller, clinically-relevant gene subsets^23^,^24^,^25^, the platforms yielded similar numbers of variant calls. To assess their accuracy, we compared each call (both the variation and its zygosity) to the NIST 2.17 and 2.19 truth sets. The sensitivity (ability to detect true positive variants), specificity (number of false positive variants per Mb), and Positive Predictive Value (PPV) for each platform are listed in Table 2. For both Single Nucleotide Variants (SNVs) and Insertion-Deletions (InDels), NextSeq achieved the highest sensitivity (99.6% of SNVs and 95.0% of InDels, respectively) followed by MiSeq (99.0% and 92.8%) and Proton (96.9% and 51.0%). PPV is nearly identical for SNVs among all the platforms. InDels are best with NextSeq (96.9%) and lowest with Proton (92.2%). After accounting for coverage differences, the NextSeq and MiSeq are nearly equivalent in performance. The apparent sensitivity with the combined platforms is as high as 99.88% for SNVs in the NIST 2.19 consensus regions. The sensitivity across non-NIST regions will likely be less due to lower coverage in many of those regions. Based on the number of exons with low coverage, the true sensitivity may be less than 98%.

Rare variants are the most relevant to clinical sequencing and detecting them can be more challenging because analysis programs are not tuned to them. To determine the sensitivity for rare variants, the protein-coding NIST regions were also examined in the ExAC database^26^. NA12878 variants detected in ExAC with a population frequency of <1% were used as the truth set. Sensitivity for detecting such variants was somewhat less than for all variants with the NextSeq detecting 98.23% of the 525 rare SNVs and 66.21% of the 23 rare InDels and the Proton detecting 96.45% and 26.09%, respectively. Combining the platforms provided sensitivities of 99.43% and 66.21%. The low number of variants in these categories limits the precision with which these values can be determined.

To determine the impact of combining two orthogonal sequencing platforms on accuracy, variant calls from the platforms were compared. Because the format of VCFs from the platforms is different and calling multi-nucleotide variants can generate multiple different but equivalent names for variants, algorithms were generated at Claritas to carry out the combination of two distinct VCF files (Combinator). We found that the overall concordance between calls was extremely high. In variant calls from three independent replicates of NA12878, nearly 95% of variants are called identically on the two platforms (a total of 49,167 orthogonally concordant variant calls over three replicates in the NIST region of RefSeq + /−10 bp).

When compared to the NIST truth set, nearly all variants matched (>99.99%). Only four variants were discordant with NIST 2.17 (Described in detail in Supplementary Discussion). All were subjected to Sanger sequencing in an attempt to disambiguate these results. Sanger sequencing confirmed that three of these four variants did not match the NIST 2.17 truth set. Inspection of a newer version (NIST 2.19) revealed that these three variants had been removed, indicating that others had also found issues at these positions. Eight additional apparent false positives were found in v2.19 but these were all confirmed to be artifacts by Sanger with 7/8 arising from v2.19 issues with properly deconvoluting multinucleotide variants. The remaining variant was found in both NIST versions and just barely passed the threshold for NextSeq coverage (8 reads, Supplementary Table 1). This single real FP among the total of 49,167 variants called yields a final Positive Predictive Value (PPV) of 99.998% for the orthogonally confirmed variants (Table 3). Raising the coverage depth threshold from 8 to 10 would eliminate the single FP and raise the PPV to 100%. However, this change would come at a sensitivity cost (approximately 0.3% fewer NextSeq TPs). The single FP is actually not a failure to detect a variant but rather an error in zygosity with the heterozygous position incorrectly called homozygous alternate.

We analyzed the remaining variants that were not orthogonally concordant on the two platforms. Less than 5% of all variants are SNVs or InDels that are called only on the NextSeq or only on the Proton. Singleton NextSeq calls have PPV~95% regardless of whether there is Proton coverage or not (Table 3). As a result, we classify them as Likely True Positives. Singleton Proton calls have a similar PPV (and classification) when there is no NextSeq coverage but significantly worse PPV when there is NextSeq coverage but there is no call or a different call so these situations are separated. Most of these variants are NextSeq-specific with the number of TPs and FPs arising from NextSeq/Proton shown in parentheses below the total. Fewer than 1% of SNVs and InDels are either a singleton NextSeq NoPass call or a singleton Proton call opposite a different NextSeq call. These have PPV~20%. We classify this small set of variants as Likely False Positives. The number of TPs and FPs arising from NextSeq/Proton are also shown in parentheses below the total. Even though we did not observe a difference in the quality of the OC variants made with NextSeq Pass versus the 134 NoPass calls, their potential for lower quality calls led us to categorize them separately. We classify them as Reliable (PPV = 100%). Prior to reporting a Reliable variant, we think it wise to inspect or further confirm them.

When multiple experiments are compared, we found that the variant classifications remain stable across replicate sequencing runs. As shown in Table 4, nearly 99% of Orthogonally Confirmed variants found in one blood sample were also OC in a replicate blood sample from the same donor. Variants identified initially as Likely TP were found to repeat as Likely TP, Reliable or OC at a rate of ~92%. In contrast, the less certain variants identified as Likely FP were categorized as Likely FP again less than one third of the time and not called at all in the second run >50% of the time. Very similar results were observed across all categories with technical replicates of the same DNA. This high level of reproducibility lends confidence to these classifications.

In order to provide maximum flexibility for patients providing biological fluids for DNA extraction, we have examined multiple sample collection methods. DNA collected from cell lines, from blood, and from saliva all performed identically in our hands (Table 4). While sensitivity and specificity could not be determined based on the lack of truth sets, the rate of orthogonal confirmation and the number of variants identified was indistinguishable between blood and saliva using clinically validated methods.

## Discussion

Today, the genetic basis for more than half of the >7000 known Mendelian disorders has been elucidated and the pace of genetic discoveries continues unabated^27^. The benefits of broad genetic testing of patients for previously undiagnosed diseases for the patient are clear^7^. However, the quality and costs of such testing and reimbursement for them have been problematic. WES provides cost-efficient identification of clinically-relevant variants that eliminates the high expense of testing only a few genes at a time and the resultant lengthy diagnostic odysseys^28^,^29^. We have demonstrated here that the use of orthogonal DNA selection and sequencing methodologies provides better sensitivity than standard WES and additionally allows immediate confirmation of ~95% of all variants. This improves turnaround time and eliminates the need and cost for most subsequent Sanger confirmation.

While Sanger sequencing is generally considered the gold standard for confirmation, it is subject to the same amplification and repeat-based artifacts that can afflict NGS technologies. For example, primer or other allele-specific effects can cause selective amplification of one allele. We found that G > A variants in very GC-rich amplicons caused differential amplification efficiency in both Sanger and NGS methods. Other sequences can cause other problems when unusual DNA structures are created^30^. The Sanger-based confirmation of three NA12878 variants could have led to two errors if the results were taken at face value (Supplementary Discussion). These difficulties highlight the issue that even the “gold standard” sequencing technology is error-prone and subject to artifacts.

In addition to the immediate confirmation of ~95% variants and the high accuracy of OC variants, another key benefit to the parallel exome sequencing is the increased sensitivity due to the overlapping regions that are covered by each platform. Because the NIST reference is biased for regions that are most easily sequenced, results as shown in Table 2 can be deceptive and overestimate the true sensitivity of both platforms. The singly covered regions allow a greater percentage of variants to be identified and subsequently confirmed by other methods. There are thousands of variants in the exome that are detected only by the NextSeq or the Proton. These variants require confirmation prior to clinical reporting but they would not have been reported at all if only the NextSeq or only the Proton had been used exclusively. By using orthogonal and complementary technologies, we are able to quickly confirm variants at a genome-wide scale and provide improved sensitivity for detecting potentially pathogenic variants.

No matter which method of sequencing is chosen, clinical quality data should be confirmed prior to reporting^10^. At the genome-wide level, this is a large burden that is sometimes addressed by prioritizing different variant calls based on pathogenicity and/or call quality. The more confident one wants to be in assuring call accuracy, the more variants require individual attention and likely Sanger sequencing. Use of orthogonal NGS eliminates virtually all needs for prioritization of variants for subsequent confirmation. While the initial cost of sequencing two exomes is higher, the ultimate savings in confirmatory sequencing as well as the improved sensitivity makes up for that expense. Sanger sequencing costs vary significantly from lab to lab but, in our hands, a single Proton exome can be prepared and sequenced for about the same cost as 25–50 individual Sanger reactions that each require custom primers. Furthermore, choosing which variants to confirm via Sanger requires waiting for the initial analysis to be completed so the inclusion of the orthogonal Proton sequencing run results in a faster turnaround time in addition to the potential for lower cost.

Speed of results is also frequently an issue. Ion Torrent systems detect pH changes electronically so are typically faster than laser/CCD camera-based Illumina sequencing systems. The various Illumina instruments have different speeds and output yields and thus could potentially have different performance as well. However, there are times where the more rapid sequencing time and lower computational requirements would be advantageous for returning results more quickly and the slight reduction in sensitivity caused by the faster but lower output of the MiSeq compared to the NextSeq would be preferable, especially since much of it would be compensated by Proton coverage. In some cases, the need for extreme speed may override cost and accuracy considerations. We have not yet attempted to minimize handling times but, in our hands, DNA extraction, library preparation and DNA sequencing require about 44 hrs of clock time and just over 7 hrs of hands-on time for the Illumina NextSeq while the Proton requires only half that for both. Reagent costs can vary significantly based on volumes purchased but we find that reagents for extraction through sequencing are about twice as much for the NextSeq compared to the Proton.

Frequently, providers wish to test a defined gene list known to be associated with a patient’s disease. When defining a gene list for a particular phenotype-driven investigation of clinically relevant variants, the existing literature is used for determining inclusion. With the continuing pace of novel discoveries, any defined gene list will not include new findings so any novel genes would lie outside the region of interest. Pathogenic variants in poorly characterized genes or novel genes would not be identified in such lists. Additionally, phenotypes or symptoms can change over time which could also affect the list of genes for which testing is desired. The orthogonal approach has the advantage of providing high sensitivity and immediate confirmation of nearly all variants on any gene list while retaining the ability to expand to the whole exome if no pathogenic variants are found. We have found this tiered approach critical with a number of clinical cases where the pathogenic variants were not identified in the initial analysis of the pre-specified gene list. However, convincing candidate variants were identified when the gene list was expanded and the remainder of the exome was examined (data not shown). The improved speed and data quality should translate into improved diagnostic rates for patients with concomitant benefits for them and their families.

## Additional Information

**How to cite this article**: Chennagiri, N. *et al.* Orthogonal NGS for High Throughput Clinical Diagnostics. *Sci. Rep.* **6**, 24650; doi: 10.1038/srep24650 (2016).

## Supplementary Material

Supplementary Information

## Figures and Tables

**Table 1:** Targeted Regions.

Region Included (#genes)	#Bases in Region	#Bases in Region Intersected with RefSeq CDS		#Variants in Region intersected with RefSeq CDS				
				NextSeq	Proton	Both	NextSeq Unique	Proton Unique
RefSeq CDS + /−10 bp	37,561,194	37,561,194	Mean	27,349	23,539	22,367	4,982	1,173
			SD	72	203	241	247	43
NIST 2.17 consensus	2,228,189,742	28,184,815	Mean	17,745	16,799	16,399	1,345	400
			SD	11	98	141	135	45
NIST 2.19 consensus	2,215,826,661	28,061,966	Mean	17,750	16,809	16,412	1,338	397
			SD	10	100	142	134	45
Clinical Research Exome	93,162,776	36,655,779	Mean	27,349	23,198	22,367	4,982	831
			SD	72	196	241	247	48
AmpliSeq Exome	57,742,646	35,894,097	Mean	25,961	23,539	22,367	3,595	1,173
			SD	52	203	241	252	43
ACMG Secondary (56)	224,829	224,829	Mean	126	120	118	8	2
			SD	0	0	1	1	1
Sudden Cardiac Death (103)	426,017	426,017	Mean	191	170	168	23	1
			SD	1	1	1	2	1
Newborn Screening (525)	1,424,598	1,424,598	Mean	841	771	755	86	16
			SD	1	6	8	9	2

**Table 2:** Sensitivity and specificity of sequencing platforms.

Type	SENS	RefSeq ∩ NIST v2.17 ∩ CRE ∩ AmpliSeq						RefSeq ∩ NIST v2.19 ∩ CRE ∩ AmpliSeq						
		SPEC (FP/MB)	PPV	#FPs	#TPs	#TNs	#FNs	SENS	SPEC (FP/MB)	PPV	#FPs	#TPs	#TNs	#FNs
Illumina MiSeq								Illumina MiSeq						
SNV	98.95%	1.78	99.71%	49	16587	27544203	176	98.99%	1.75	99.71%	48	16585	27431566	168
Indel	91.94%	0.65	96.07%	18	441	27544203	39	92.79%	0.53	96.90%	15	459	27431566	36
All	98.76%	2.43	99.61%	67	17028	27544203	214	98.82%	2.29	99.63%	63	17044	27431566	204
Illumina NextSeq								Illumina NextSeq						
SNV	99.56%	1.84	99.70%	51	16705	27544186	74	99.60%	1.85	99.70%	51	16704	27431546	67
Indel	94.23%	0.61	96.44%	17	452	27544186	28	95.00%	0.55	96.90%	15	469	27431546	25
All	99.41%	2.44	99.61%	67	17157	27544186	102	99.47%	2.39	99.62%	66	17173	27431546	91
Proton Filtered								Proton Filtered						
SNV	96.85%	2.65	99.55%	73	16250	27544161	529	96.89%	2.66	99.55%	73	16251	27431518	521
Indel	51.79%	0.80	91.87%	22	251	27544161	233	50.96%	0.78	92.20%	21	254	27431518	244
All	95.62%	3.45	99.43%	95	16501	27544161	756	95.61%	3.44	99.43%	94	16505	27431518	759
NextSeq/Proton Combined								NextSeq/Proton Combined						
SNV	99.86%	4.31	99.30%	119	16757	27544096	25	99.88%	4.31	99.30%	118	16753	27431457	20
Indel	94.31%	1.40	92.15%	39	453	27544096	27	95.01%	1.32	92.83%	36	469	27431457	25
All	99.71%	5.71	99.09%	157	17210	27544096	51	99.74%	5.64	99.11%	155	17222	27431457	45

**Table 3:** Variant categories in orthogonal sequencing.

Category	Region Analyzed: RefSeq ∩ NIST 2.17 ∩ CRE ∩ AmpliSeq			
	% of Total	# FP	# TP	PPV
Orthogonally Confirmed – NextSeq Pass call matches Proton call	94.4%	1	49167	99.998%
Reliable - NextSeq NoPass or filtered call matches Proton call	0.3%	0	134	100.00%
Likely True Positives - Singleton NextSeq call or Singleton Proton call with no NextSeq coverage	4.6%	124(103/21)	2249(2129/120)	94.77%
Likely False Positives - Singleton NextSeq NoPass or Singleton Proton call with NextSeq coverage	0.8%	346(97/249)	79(18/61)	18.59%

**Table 4:** Reproducibility of orthogonal sequencing category.

	Orthogonally Confirmed	Reliable	Likely TP	Likely FP	Not called
Orthogonally Confirmed	16030	37	226	9	5
Reliable	27	7	12	0	0
Likely TP	226	7	611	11	34
Likely FP	9	2	8	45	128
Not called	0	0	40	108	0
Saliva vs. Blood					
Orthogonally Confirmed	16581	40	143	11	1
Reliable	25	12	12	1	0
Likely TP	234	15	435	12	42
Likely FP	8	0	8	46	137
Not called	4	0	35	83	0
Blood vs. Blood					
Orthogonally Confirmed	16674	40	148	9	1
Reliable	10	16	8	0	0
Likely TP	156	11	428	16	37
Likely FP	8	0	6	48	85
Not called	4	0	43	80	0

## References

[b1] de Ligt J. *et al.* Diagnostic exome sequencing in persons with severe intellectual disability. N Engl J Med 367, 1921–1929 (2012). 23033978 10.1056/NEJMoa1206524

[b2] Farwell K. D. *et al.* Enhanced utility of family-centered diagnostic exome sequencing with inheritance model-based analysis: results from 500 unselected families with undiagnosed genetic conditions. Genet Med 17, 578–586 (2015). 25356970 10.1038/gim.2014.154

[b3] Iglesias A. *et al.* The usefulness of whole-exome sequencing in routine clinical practice. Genet Med 16, 922–931 (2014). 24901346 10.1038/gim.2014.58

[b4] Srivastava S. *et al.* Clinical whole exome sequencing in child neurology practice. Ann Neurol 76, 473–483 (2014). 25131622 10.1002/ana.24251

[b5] Yang Y. *et al.* Clinical whole-exome sequencing for the diagnosis of mendelian disorders. N Engl J Med 369, 1502–1511 (2013). 24088041 10.1056/NEJMoa1306555 PMC4211433

[b6] Zhu X. *et al.* Whole-exome sequencing in undiagnosed genetic diseases: interpreting 119 trios. Genet Med 17, 774–781 (2015). 25590979 10.1038/gim.2014.191 PMC4791490

[b7] Directors A. B. o. Clinical utility of genetic and genomic services: a position statement of the American College of Medical Genetics and Genomics. Genet Med 17, 505–507 (2015). 25764213 10.1038/gim.2015.41

[b8] Mardis E. R. Next-generation sequencing platforms. Annu Rev Anal Chem (Palo Alto Calif) 6, 287–303 (2013). 23560931 10.1146/annurev-anchem-062012-092628

[b9] Thompson J. F. & Milos P. M. The properties and applications of single-molecule DNA sequencing. Genome Biol 12, 217 (2011). 21349208 10.1186/gb-2011-12-2-217 PMC3188791

[b10] Rehm H. L. *et al.* ACMG clinical laboratory standards for next-generation sequencing. Genet Med 15, 733–747 (2013). 23887774 10.1038/gim.2013.92 PMC4098820

[b11] Asan *et al.* Comprehensive comparison of three commercial human whole-exome capture platforms. Genome Biol 12, R95 (2011). 21955857 10.1186/gb-2011-12-9-r95 PMC3308058

[b12] Bodi K. *et al.* Comparison of commercially available target enrichment methods for next-generation sequencing. J Biomol Tech 24, 73–86 (2013). 23814499 10.7171/jbt.13-2402-002 PMC3605921

[b13] Chilamakuri C. S. *et al.* Performance comparison of four exome capture systems for deep sequencing. BMC Genomics 15, 449 (2014). 24912484 10.1186/1471-2164-15-449 PMC4092227

[b14] Lelieveld S. H., Spielmann M., Mundlos S., Veltman J. A. & Gilissen C. Comparison of Exome and Genome Sequencing Technologies for the Complete Capture of Protein-Coding Regions. Hum Mutat 36, 815–822 (2015). 25973577 10.1002/humu.22813 PMC4755152

[b15] Meienberg J. *et al.* New insights into the performance of human whole-exome capture platforms. Nucleic Acids Res 43, e76 (2015). 25820422 10.1093/nar/gkv216 PMC4477645

[b16] Samorodnitsky E. *et al.* Evaluation of Hybridization Capture Versus Amplicon-Based Methods for Whole-Exome Sequencing. Hum Mutat 36, 903–914 (2015). 26110913 10.1002/humu.22825 PMC4832303

[b17] Shigemizu D. *et al.* Performance comparison of four commercial human whole-exome capture platforms. Sci Rep 5, 12742 (2015). 26235669 10.1038/srep12742 PMC4522667

[b18] Warr A. *et al.* Exome Sequencing: Current and Future Perspectives. G3 (Bethesda) 5, 1543–1550 (2015). 26139844 10.1534/g3.115.018564 PMC4528311

[b19] Zhang G. *et al.* Comparison and evaluation of two exome capture kits and sequencing platforms for variant calling. BMC Genomics 16, 581 (2015). 26242175 10.1186/s12864-015-1796-6 PMC4524363

[b20] DePristo M. A. *et al.* A framework for variation discovery and genotyping using next-generation DNA sequencing data. Nat Genet 43, 491–498 (2011). 21478889 10.1038/ng.806 PMC3083463

[b21] Carson A. R. *et al.* Effective filtering strategies to improve data quality from population-based whole exome sequencing studies. BMC Bioinformatics 15, 125 (2014). 24884706 10.1186/1471-2105-15-125 PMC4098776

[b22] Zook J. M. *et al.* Integrating human sequence data sets provides a resource of benchmark SNP and indel genotype calls. Nat Biotechnol 32, 246–251 (2014). 24531798 10.1038/nbt.2835

[b23] Green R. C. *et al.* ACMG recommendations for reporting of incidental findings in clinical exome and genome sequencing. Genet Med 15, 565–574 (2013). 23788249 10.1038/gim.2013.73 PMC3727274

[b24] Li M. H. *et al.* Utility and limitations of exome sequencing as a genetic diagnostic tool for conditions associated with pediatric sudden cardiac arrest/sudden cardiac death. Hum Genomics 9, 15 (2015). 26187847 10.1186/s40246-015-0038-y PMC4506570

[b25] Saunders C. J. *et al.* Rapid whole-genome sequencing for genetic disease diagnosis in neonatal intensive care units. Sci Transl Med 4, 154ra135 (2012). 10.1126/scitranslmed.3004041 PMC4283791 23035047

[b26] Exome Aggregation Consortium *et al.* Analysis of protein-coding genetic variation in 60,706 humans. *bioRxiv* (2015).

[b27] Chong J. X. *et al.* The Genetic Basis of Mendelian Phenotypes: Discoveries, Challenges, and Opportunities. Am J Hum Genet 97, 199–215 (2015). 26166479 10.1016/j.ajhg.2015.06.009 PMC4573249

[b28] Deverka P. A. & Dreyfus J. C. Clinical integration of next generation sequencing: coverage and reimbursement challenges. J Law Med Ethics 42 Suppl 1, 22–41 (2014). 25298289 10.1111/jlme.12160 PMC5108048

[b29] van Nimwegen K. J. *et al.* The diagnostic pathway in complex paediatric neurology: a cost analysis. Eur J Paediatr Neurol 19, 233–239 (2015). 25604808 10.1016/j.ejpn.2014.12.014

[b30] Lira M. E., Lloyd D. B., Hallowell S., Milos P. M. & Thompson J. F. Highly polymorphic repeat region in the CETP promoter induces unusual DNA structure. Biochim Biophys Acta 1684, 38–45 (2004). 15450208 10.1016/j.bbalip.2004.06.002

