# Supplementary material for: Orthogonal NGS for High Throughput Clinical Diagnostics
Source: Sci Rep. 2016 Apr 19;6:24650. doi: 10.1038/srep24650 (PMC4836299; doi:10.1038/srep24650)

Orthogonal NGS for High Throughput Clinical Diagnostics

Niru Chennagiri[1], Eric J. White[1], Alexander Frieden[1], Edgardo Lopez[1], Daniel S. Lieber[1], Anastasia Nikiforov[1], Tristen Ross[1], Rebecca Batorsky[1], Sherry Hansen[1], Va Lip[1], Lovelace J. Luquette[2], Evan Mauceli[1], David Margulies[1, 2, 3], Patrice M. Milos[1], Nichole Napolitano[1], Marcia M. Nizzari[1], Timothy Yu[1], John F. Thompson[1]

[1] Claritas Genomics, Cambridge MA
[2]Harvard Medical School, Boston MA
[3]Boston Children's Hospital, Boston MA

Please direct all correspondence to:
John F. Thompson, Claritas Genomics, 99 Erie St, Cambridge MA 02139
John.thompson@claritasgenomics.com
617-553-5815

Supplementary Discussion

As discussed above, Sanger sequencing was carried out on all orthogonally confirmed variants that appeared as putative false positives in the initial screening of the NIST NA12878 reference sequence. The initial list was subsequently filtered using supplemental hard thresholds for DP and GQ. Three variants (Chr9:15623462/rs405352, Chr15:45445692/rs1648303, Chr19:4511350/ rs7259721) were identified in our studies that did not match the specific variants listed in the NIST 2.17 VCF reference. These regions were absent from the NIST 2.19 VCF. When we Sanger sequenced these variants, we found discordances with the NIST 2.17 reference. These OC variants will not be discussed further as the calls in the NIST 2.17 reference were incorrect as detected by us and others.

Chr19:7708214/rs2303115 was identified using standard GATK filters without DP and GQ. This variant was filtered out by the coverage threshold but Sanger sequencing was attempted anyway. It was not able to be sequenced using standard Sanger conditions, but GC-rich sequencing conditions showed it was heterozygous, in agreement with the reference and two NGS runs. As can be seen by the low coverage in the orthogonally confirmed run (7 reads each in NextSeq and Proton), this is simply a case where low coverage contributed to an errant call and reinforced the need for coverage thresholds to prevent such random statistical anomalies.

The other two variants were not as straightforward and as we were unable to generate quality sequence data at these positions using standard conditions. Chr22:38221032/rs571021482 was not called on NGS2 and was called differently on NGS1 (CT) versus NGS3 (TT). Because standard sequencing conditions yielded no data, three additional sequencing conditions that favored GC-rich DNA were attempted with both forward and reverse primers for the two DNA sources (NIST and Coriell) for a total of 12 NA12878 sequencing reactions. In one condition, two forward reads and one reverse could be clearly called homozygous T, in agreement with the orthogonally confirmed call and different than the reference. The fourth reaction yielded no data. However, the other two sequencing conditions yielded 8/8 reactions that were clearly heterozygous, in agreement with the non-orthogonally confirmed NGS run and the reference call (Supplementary Figure 2A). To help disambiguate these results, the parental samples were examined. One parent, NA12891, yielded the same results as NA12878, either TT or CT, depending on the sequencing conditions. The other parent, NA12892, yielded homozygous C in two sequencing conditions and failed to yield any sequence in the condition that generated homozygous T calls in the other samples. Of note, there is one additional heterozygous CT polymorphism (Chr22:38220964/rs8138029) in the heterozygous rs571021482 Sanger reads. In the homozygous rs571021482 Sanger reads, both positions are homozygous, suggesting that the 3 Sanger reads and the orthogonally confirmed NGS reads suffered from allele dropout in which the difficulties of amplification allowed the slightly more AT-rich allele to amplify selectively over the slightly more GC-rich allele. The fact that there are two apparent changes separated by 68 bp lowers the likelihood that they are *de novo* variants. Because different primers were used in the NGS and Sanger reactions, it was unlikely to have been

caused by variants in the primer regions. Thus, this variant is likely to be a false positive caused by amplification-dependent allele dropout. Unfortunately, in a real clinical situation, it is quite possible that Sanger sequencing would actually lead to "confirmation" of the wrong call since results are dependent on the sequencing conditions used.

Chr19:3054089/rs2302301 does not pass depth filters (2-4 reads on the two NextSeq runs) but it is instructive to analyze this variant as it reinforces the issues seen with Chr22:38221032/rs571021482. With rs2302301, 2 of 3 NGS runs indicate an orthogonally confirmed TT call, different than the reference and the other NGS run. All 12 Sanger sequencing runs also appeared as TT, in agreement with the two orthogonally-confirmed NGS runs. More than half the runs achieved the maximum quality score that would normally lead one to believe that the reference is wrong. When the parental DNA was examined, NA12891 was clearly homozygous TT while NA12892 was clearly homozygous CC. If the likelihood of *de novo* mutation is considered low, the problem of selective amplification is again the most likely culprit. Indeed, a trace C signal can be seen in all Sanger reactions (Supplementary Figure 2B). In standard sequencing conditions, no readable sequence was observed. In the GC-rich conditions, both alleles could be amplified efficiently but the T allele could do so much more efficiently than the C allele, leading to allele dropout when both are present. Thus, in a clinical situation, this variant would have failed the coverage filter and submitted for Sanger confirmation. Sanger error would have caused it to be misreported. These instances of allele dropout highlight the difficulties in achieving 100% accuracy in a highly complex genome, no matter which technology is used.

Supplementary Tables and Figures

Supplementary Table 1

Detection of putative false positives in orthogonal sequencing runs

Putative FP that Passes DP/GQ Threshold

Position/dbSNP	Platform	NGS 1	NGS 2	NGS 3	Ref	Alt	Correct	NIST	Sanger
Chr22:38221032 rs571021482	Orthogonal Call:	CT	No Call	TT	C	T	CT	CT	CT/TT
	NextSeq	10/9	6/2	0/8					
	Proton	0/0	0/0	0/21					

Putative FPs that Do Not Pass DP Threshold

Position/dbSNP	Platform	NGS 1	NGS 2	NGS 3	Ref	Alt	Correct	NIST	Sanger
Chr19:7708214 rs2303115	Orthogonal Call:	AA	GA	GA	G	A	GA	GA	GA
	NextSeq	0/7	4/5	3/6					
	Proton	0/7	1/4	1/3					
Chr19:3054089 rs2302301	Orthogonal Call:	TT	CT	TT	C	T	CT	CT	TT
	NextSeq	0/4	1/2	0/2					
	Proton	3/51	2/22	1/71					

Supplementary Figure 1

GC content vs. coverage

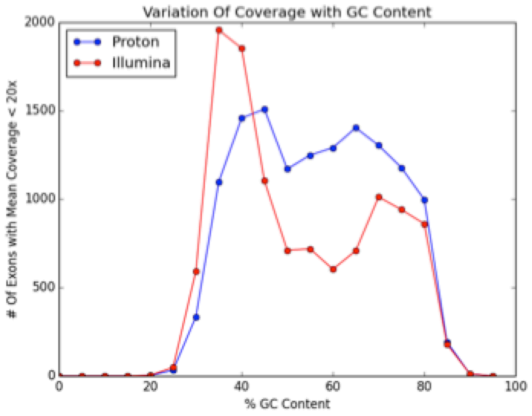

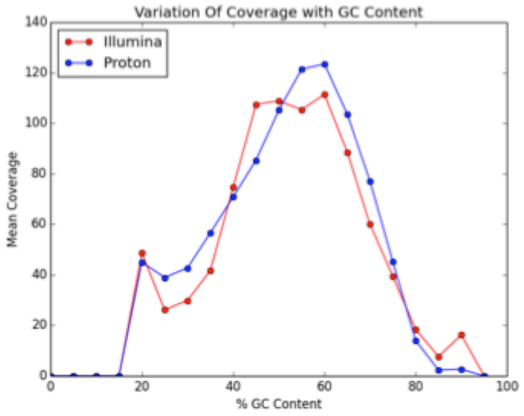

Supplementary Figure 2
Sanger sequencing traces

A)

GALR3 rs571021482 – NGS calls: CT/TT/NC

Standard Sanger Conditions: No readable sequence

Sequencing Condition #1 (3 x TT, 1 x NC) Sequencing Condition #2/3 (8 x CT)

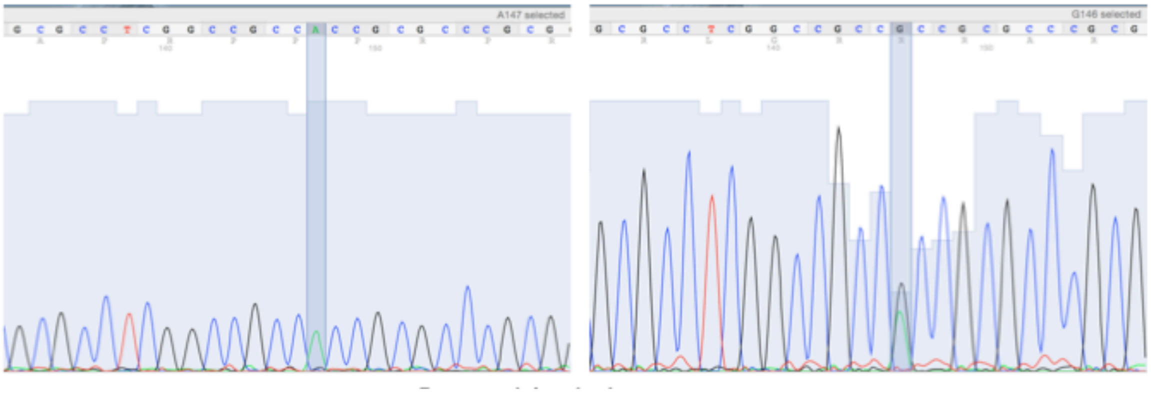

B)

AES rs2302301 – NGS calls: TT/TT/CT

Standard Sanger Conditions: No readable sequence

Sequencing Condition #1 - 4 x TT Forward Sequencing Condition #2/3 - 8 x TT Reverse

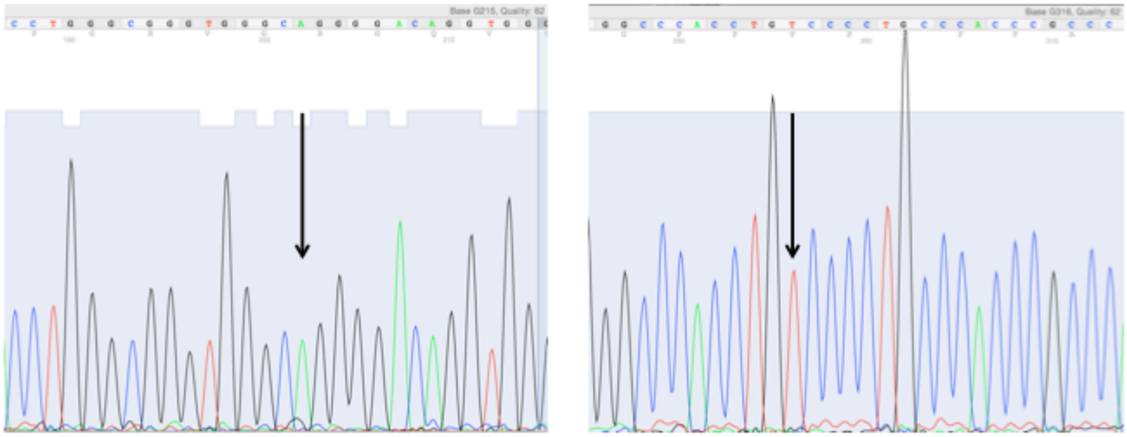

Supplement: Supplementary Information [file srep24650-s1.pdf]
